# Medical Library for the State

**Published:** 1893-10

**Authors:** 


### MEDICAL LIBRARY FOR THE STATE.

---

Dr. Robert Battey, of Rome, has donated his medical library to the State. Following is his letter to Governor Northen:

ROME, GA., September 18, 1893.

*Hon. W. J. Northen, Governor:*

In the well-nigh half century which I have devoted to the study of the science of medicine and its collateral branches—a study now drawing to a close—I have been much impressed with the lack of some large reference library anywhere within my reach. With such assistance I can but feel that my own single life would have borne more and better fruit in the service of our people. We ought to have in Georgia such a library. The medical men of Georgia need it; the courts and the lawyers of Georgia need it; the legislature of Georgia needs it. It ought to be placed in a separate alcove in the State library at the State capitol, where it would be of easy access to all, and would be properly cared for. I believe that this can all be done at a very small expenditure of the public fund.

Through your excellency as governor, I offer to the State my private library consisting of several hundred—perhaps a thousand—volumes, to be delivered in Atlanta free of cost for a beginning after the proposed alcove in the State library is set apart, and will add to it as my life may be prolonged other similar contributions. I believe that other physicians in Georgia, and especially those retired from active practice, will aid the collection by donations from year to year, until this department of the State library will have grown to be of great public utility.

I am, governor, respectfully your obedient servant,

ROBERT BATTEY.

This valuable gift of Dr. Battey should be but the beginning of a large reference medical library. There is not in the State such a library where medical men can find the data and literature necessary to the preparation of important papers. Possibly one reason why Southern doctors write so little is that the literature of a given subject is not accessible. We think the Georgia doctors will be pleased to see a large medical library in the State capitol. The movement begun by Dr. Battey deserves our hearty encouragement, and we hope the nucleus which he has established will receive such additions as will secure for the State a medical library complete in all departments.

---

We have received a copy of the "Transactions" of the last meeting of the Medical Association of Georgia. The volume is larger than usual and contains a good list of excellent papers, some of which have already appeared in these pages. We are sorry, however, to note that one of the articles is nothing more than a copy of a few selected paragraphs from the works of Ultzmann and Gross. To illustrate:

TRANSACTIONS, PAGE 116.

The spermatozoa of normal semen consists of an oval or flattened, pear-shaped, shovel-like head, and a long, thread-like end which is divided into a middle piece and a tail. The middle piece and tail should be at least ten times the length of the head. The spermatozoa should be present in large numbers, and still show movement at least twelve hours after ejaculation.

ULTZMANN'S "GENITO-URINARY NEUROSES," PAGE 85.

The spermatozoa of normal semen consists of an oval or flattened, pear-shaped, shovel-like head, and a long, thread-like end which is divided into a middle piece and a tail. The middle piece and tail should be at least ten times the length of the head. The spermatozoa should be present in large numbers, and should show movement at least twelve hours after evacuation.

This paper was not read at the Association, and it is to be regretted that it found a place in the published transactions. This is not the first time that we have commented in these pages on just such cases as this. Plagiarism is generally detected, sooner or later, and it is the part of the journals to call attention to the offence wherever found. We regret that the present example is so near home.

